# Impact of Ramadan Diurnal Intermittent Fasting on Hypoglycemic Events in Patients With Type 2 Diabetes: A Systematic Review of Randomized Controlled Trials and Observational Studies

**DOI:** 10.3389/fendo.2021.624423

**Published:** 2021-03-08

**Authors:** Dana Abdelrahim, MoezAlIslam E. Faris, Mohamed Hassanein, Ayman Z. Shakir, Ayesha M. Yusuf, Aljohara S. Almeneessier, Ahmed S. BaHammam

**Affiliations:** ^1^ Department of Nutrition and Food Technology, Faculty of Agriculture, The University of Jordan, Amman, Jordan; ^2^ Department of Clinical Nutrition and Dietetics, College of Health Sciences/Research Institute for Medical and Health Sciences (RIMHS), University of Sharjah, Sharjah, United Arab Emirates; ^3^ Endocrine Department, Dubai Hospital, Dubai Health Authority, Dubai, United Arab Emirates; ^4^ Department of Family and Community Medicine, King Saud University, Riyadh, Saudi Arabia; ^5^ Department of Medicine, College of Medicine, The University Sleep Disorders Center, King Saud University, Riyadh, Saudi Arabia

**Keywords:** caloric restriction (CR), diet, hypoglycemia, glucose, lifestyle, mealtime

## Abstract

Ramadan is the 9th month of the lunar calendar during which Muslims abstain from food and drink between dawn and sunset for 30 consecutive days. Ramadan fasting is observed by all healthy Muslim adults, as well many Muslims with type 2 diabetes (T2DM). Hypoglycemic events (HE) are a serious complication associated with diabetes management and are associated with increased cardiovascular disease risk. Conflicting results have been reported concerning the incidence of HE among people with T2DM observing Ramadan fasting. This review summarizes available scientific evidence on the occurrence of HE and the effects of different moderators on the incidence of HE among patients with T2DM during Ramadan. We conducted a systematic review of available observational studies and randomized controlled trials (RCTs) for patients with T2DM who fasted during Ramadan, with HE as the primary outcome. Ten databases were searched for relevant studies from inception until October 31, 2020. In total, 68 studies (35 RCTs and 33 observational studies) met the inclusion criteria. Non-sulfonylureas hypoglycemic medications showed superior effects in lowering the incidence of HE over sulfonylureas hypoglycemic medications. Variable moderators were associated with experiencing HE during Ramadan in both observational studies and RCTs, including sex, geographical location, body anthropometric indicators, season, dietary behaviors, fasting duration, time since diagnosis, and pre-fasting education. This comprehensive systematic review covered the largest number of observational and clinical studies investigating the impact of Ramadan on HE among patients with T2DM. The study highlights the significance of different moderators that influence the effect of Ramadan fasting on HE, including dietary behaviors, fasting time duration, sex, season, country, pre-fasting education, age, and time since diagnosis. The study also highlighted the impact of different hypoglycemic medications on HE and noted the superiority of non-sulfonylureas over sulfonylureas hypoglycemic medications in lowering the risk for hypoglycemia in people with T2DM during Ramadan fasting.

## Introduction

Ramadan is the 9th holy month of the Islamic lunar calendar. Fasting during Ramadan is an obligatory duty for all healthy adult Muslims, as stated in the Holy Quran where ALLAH says, “***O you who believe, fasting is prescribed for you as it was prescribed for those before you, that you may develop God-consciousness”*** (Surat Al-Baqarah 2:183). However, the Holy Quran exempts those who are sick, medically unfit, or traveling from fasting during the holy month: “***Yet if one among you is sick or is on a journey, [such a person shall then fast] the same number of other days*”** (Surat Al-Baqarah, 2:185). A majority of Muslim people with diabetes see this fast as a deeply meaningful, spiritual experience; therefore, most still participate, even against medical advice (1). Fasting during Ramadan dictates complete abstinence from food and drink (including water) from dawn to sunset. This encompasses a period of 10–21 h (2) depending on the geographical location and solar season that crosses with the lunar month and continues for 29–30 consecutive days. This abstinence also extends to medications used by patients who choose to fast during the holy month. This may require changes in the timing and possibly medication dose according to the dawn-to-sunset fasting time.

There are around 1.9 billion Muslims worldwide distributed across more than 200 countries and territories and accounting for 25% of the world’s population (3). The global diabetes prevalence in 2019 was estimated at 9.3% (463 million people) (4). Besides, the population-based Epidemiology of Diabetes and Ramadan 2001 study, which included 12,243 patients with diabetes from 13 Islamic countries, estimated that about 79% of patients with type 2 diabetes (T2DM) and about 43% of patients with type 1 diabetes (T1DM) fast during Ramadan (5). This means that about 70 million (50–90 million) people with diabetes worldwide may practice fasting during Ramadan. A retrospective observational study including 3,394 evaluable diabetes cases from 13 countries, found that 64% of patients reported fasting every day, and 94.2% fasted for at least 15 days (6). Given that many patients with diabetes observe fasting during Ramadan, glycemic control management for those patients is a critically important health challenge worldwide (7).

During the last few decades, medical doctors, endocrinologists, and diabetologists observing Muslim patients with diabetes have accumulated growing knowledge and clinical experience. Available research has considered various aspects of fasting during Ramadan. Research topics include clinical (8–10) and metabolic (11, 12) complications, the impact of patient education and pre-fasting preparation (13–19), medication adjustment and insulin dosing (14, 20, 21), and dietary/lifestyle modifications (21, 22) during Ramadan and how these changes impact the outcomes at the end of the fasting month.

Clinical management of intermittent fasting in patients with diabetes is receiving increased attention, including non-religious intermittent fasting practiced by these patients (23). This is because fasting in this population is associated with various risks, including hypoglycemia events (HE) or episodes, postural hypotension, dehydration, increased viscosity of the blood leading to diabetic ketoacidosis, and thrombosis (particularly in T1DM) (24, 25). HE is among the most serious changes patients with diabetes may face during fasting, and are the most challenging complications experienced in T1DM/T2DM management (26, 27). HE refers to an abnormally low concentration of glucose in the blood caused by excessive exercise, insufficient food intake, or overdosage with oral hypoglycemic agents (OHGAs) or insulin (28, 29). HE may lead to impairment of the counter-regulatory system, increased cardiovascular events (even death), and other detrimental effects. These harmful effects are more pronounced in severe HE. A prospective cohort study including 1,066 adults with T2DM aged 60–75 years reported the odds of suffering a macrovascular event were higher in patients with a history of severe HE (30). Blood glucose monitoring, recognition of HE risk factors, educational programs for healthcare professionals and patients with diabetes, and selection of appropriate regimens are key strategies to optimize glycemic control, prevent long-term complications, and minimize the risk for HE (31).

Compared with the preceding months, observation of Ramadan diurnal intermittent fasting (RDIF) by patients with T2DM has been associated with a 7.5-fold increased risk for severe HE (defined as requiring hospitalization) (5). This was supported by another study (32) that found that switching anti-hyperglycemic treatment from sulfonylureas (SU) agents to sitagliptin for Muslim patients with T2DM who fasted during Ramadan was associated with a 50% reduction in the risk for symptomatic HE. That study also reported that the incidence of symptomatic HE was as high as 20% during Ramadan in those with T2DM receiving SU treatment. In contrast, other studies did not report any adverse metabolic effects (including HE) among patients with diabetes observing RDIF (33, 34). The present systematic review explored current medical evidence on the prevalence of HE among patients with diabetes observing RDIF. This systematic review will guide physicians in their clinical decision-making, delivery of care, and developing policies regarding Ramadan fasting by patients with diabetes (35).

## Methods

### Search Strategy

Two authors (MF and DA) conducted an electronic search of 10 databases: EBSCOhost, CINAHL, Cochrane, EMBASE, PubMed/MEDLINE, Scopus, Google Scholar, ProQuest Medical, ScienceDirect, and Web of Science from 1950 until October 31, 2020. Key search terms included: “Ramadan fasting” OR “Islamic fasting” OR “Ramadan intermittent fasting” OR “Ramadan diurnal fasting” OR “Ramadan model of intermittent fasting” OR “Intermittent prolonged fasting during Ramadan” OR “Ramadan fast” OR “Recurrent circadian fasting” AND “Hypoglycemia” OR “Hypoglycaemia” OR “Hypoglycemic events” OR “Hypoglycemic episodes” AND “Diabetes mellitus” OR “Type 2 diabetes mellitus” OR “T2DM.” Additional articles were obtained by searching relevant systematic reviews and reviews, as well as hand-searching the reference lists of relevant studies. Corresponding and other authors were contacted *via* email or Research Gate to obtain missing full-text articles. As necessary, authors were contacted to obtain relevant articles and reviews and ensure that all related publications were included in this review.

### Study Selection

The *PRISMA Statement for Reporting Systematic Reviews and Meta-Analyses of Studies was used to i*dentify data sources (36). Papers were first subdivided into observational studies or clinical interventional trials involving patients with T2DM who decided to observe Ramadan fasting. The inclusion criteria for the selected observational and clinical studies were papers that: 1) were published as a primary research paper in a peer-reviewed journal; 2) included cohorts of patients with T2DM that opted to practice intermittent fasting during Ramadan; 3) reported the primary outcome of hypoglycemia or HE before, during (as the primary outcome), or after Ramadan fasting month; and 4) included male or female patients with diabetes. We included studies involving all patients with T2DM that intended to fast during Ramadan and were taking insulin or non-insulin antidiabetic agents. These agents included metformin (MTF), SU agents, meglitinides, thiazolidinediones, glucagon-like peptide (GLP)-1 receptor analogs, alpha-glucosidase inhibitors, dipeptidyl peptidase-4 (DPP-4) inhibitors, and sodium-glucose co-transport 2 inhibitors (SGLT2-I). The frequency of documented or symptomatic HE during Ramadan fasting was reported as the primary endpoint where available. HE or episodes were defined as patients’ subjective symptoms or documented blood glucose <70 mg/dl (3.9 mmol/L). Severe HE was defined as an episode of HE requiring third-party assistance or hospitalization (29).

## Results

**Figure 1:**
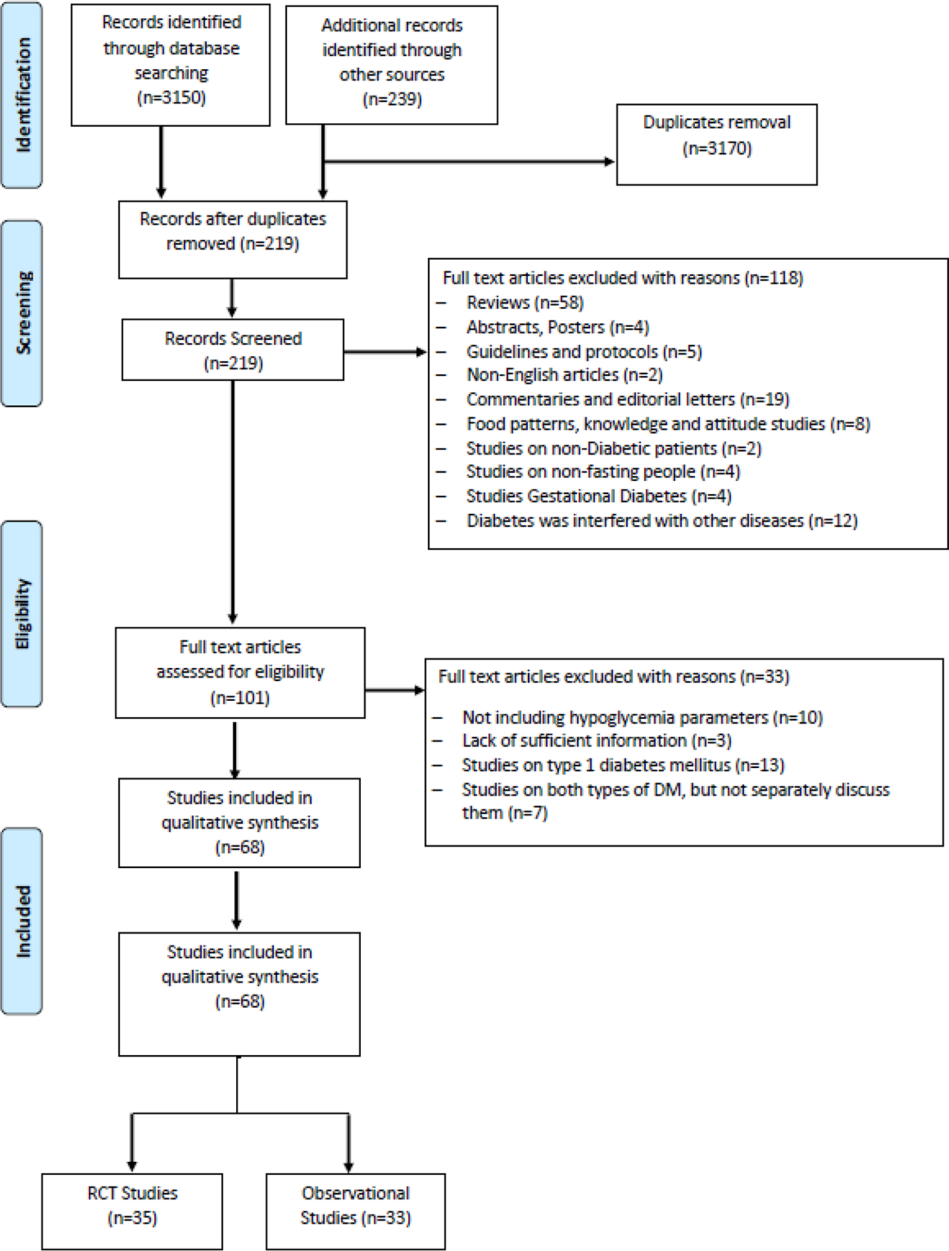
Flow chart for study selection.

Of the 3,389 studies initially retrieved, 68 studies that investigated the effects of RDIF on HE among patients with diabetes observing RDIF met the inclusion criteria. **Figure 1** presents the stages of evaluation and exclusion of the identified studies. **Table 1** summarizes the general characteristics of these studies. The extracted information included the year of publication, country/city, the season of the fasting month, fasting duration (hours), study design, number of participants and percentage of male participants, type of diabetes studied, age of participants, duration since diabetes diagnosis, type of intervention (fasting alone or with other medical/dietary/educational interventions), tested outcome(s), and results. All included studies used a pre-post model to report changes in HE.

**Table 1:** Descriptive data for included studies.

Authors, year of publication	Country (City)	Ramadan start and end dates, year	Season	Fasting period(hour/day)	Study design	Subjects (%males)	Type of DM	Age(mean ± SD)/Range	Duration of diabetes (year) (mean ± SD)/Range	Intervention	Measured outcome
Ahmedani and Ghafoor 2020 (18)	Pakistan (Karachi)	28 June– 27 July 2014	Summer	13.31	Prospective Pre and post RCT	54 (48)	Insulin-treated T2DM	54.65 ± 9.32	13.83 ± 6.27	Insulin dose adjustments Diet plan SMBG	- HbA1c (%) - Serum creatinine - HE
Cesur et al., 2007 (37)	Turkey	15 October– 13 November 2004	Autumn	12.5	Open-label, multicenter, prospective, observational	49 (59)	T2DM	33–67	6.4–4.9	Glimepiride, Repaglinide, Insulin glargine	- FBG, PBG, - HbA1c, - Fructosamine, - TC, TG, HDL, LDL
Mattoo et al., 2003 (38)	India, Pakistan, Malaysia, Singapore, Egypt, Morocco, South Africa	Varied	Varied	Varied	Open-label, multicenter, randomized, crossover RCT	151 (45.7)	T2DM	53 ± 9 (30–72)	12.5 ± 6.5 (0.3–32.9)	Insulin Lispro Mix25, Human insulin 30/70	- FBG, PBG
Bashir et al., 2018 (39)	Qatar	June. 2017	Summer	15	Prospective pilot observational	16 (91) (10 active and 6 sedentary)	T2DM	53.4 ± 6.4	15 ± 5.9	Multiple anti-diabetic agents FCGM	- HbA1c - Incidence of HE (symptomatic and asymptomatic)
Hassanein et al., 2020 (34)	Nine Asian and Middle-Eastern countries	Varied	Varied	Varied	Prospective, international, observational	1244 (54.8)	T2DM	54.1 ± 10.5	5.4 ± 5.7	Gliclazide modified-release	- The proportion of patients reporting >1 symptomatic HE. - HbA1c, - FPG - Body weight
Hassanein et al., 2019 (40)	India, Kuwait, Lebanon, and Turkey, Israel	27 May – 24 June 2017	Summer	Varied	international, multi-center, randomized, open-label, parallel-group, clinical trial RCT	21 (71)	T2DM	59.1 ± 8.7 (34–71)	7.3 ± 5.1	Ramadan fasting	- CGM with FSL-CGM
Akram et al., 1999 (41)	Egypt, Saudi Arabia, UAE, Pakistan	Varied	Varied	12–13	Open-label, randomized, crossover, multicenter RCT	70 (55.9)	T2DM treated with insulin	50.3 ± 1.1	11.6 ± 0.7	Insulin Lispro with soluble human insulin	- Total number of HE - FBG, FPG
Anwar et al., 2006 (42)	Malaysia (Kuala Lumpur)	04 October– 03 November 2005	Autumn	13	5-month open-label, parallel-group, comparative RCT	Repaglinide (17, 47.1) Glimepiride (21, 38.1)	T2DM	Repaglinide (5, 35–65) Glimepiride (49, 30–74)	Repaglinide (7, 2–20) Glimepiride (4, 2–18)	Repaglinide or glimepiride treatment	- Total number of HE
Abdelgadir et al., 2019 (43)	UAE (Dubai)	06 June– 05 July 2016	Summer	16–17	Prospective interventional RCT	SGLT2I (49, 34.8) Non-SGLT2I (46, 38.8)	T2DM	SGLT2I (57.5 ± 9.1) Non-SGLT2I (55.4 ± 9.4)	NR	GLT2I along with insulin, using FGMS	- Total number and duration of HE
Aldawi et al., 2018 (44)	UAE (Ajman)	26 May – 24 June.2017	Summer	16–17	Prospective observational	33 (73)	T2DM	55.0 ± 9.8	10.4 ± 6.8	Ramadan fasting, education, CGM	- HbA1c - FPG - HE
Hassanein et al., 2020 (45)	Kuwait, Qatar, Saudi Arabia, UAE, Jordan, Lebanon, Turkey, Egypt, India, Pakistan, Canada	Varied	Varied	Varied	Prospective, observational	430 (51.7)	T2DM	54.4 ± 11.0	The median (Q1: Q3) duration of diabetes was 9.1(5.0:15.0) years and almost half (230 [46.7%]) of the participants had a T2DM duration of ≥10 years	Participants with T2DM treated with Gla-300 in pre-Ramadan, Ramadan, and post-Ramadan periods	- symptomatic documented HE
Aravind et al., 2011 (46)	India, Malaysia, Israel, UAE, and Saudi Arabia	Varied	Summer	Varied	Prospective, observational	1397 (53)	T2DM	54	NR	Ramadan fasting SU-treatment for diabetes Glimepiride, Gliclazide, or Glibenclamide with or without MTF	- Symptomatic HE
Aravind et al., 2012 (32)	Malaysia	28 June– 10 October.2010	Summer	12.13	Multicenter, pragmatic, randomized RCT	870 (47)	T2DM	51	NR	Sitagliptin and SU (Glimepiride, Gliclazide, or Glibenclamide) with or without MTF	- Incidence of symptomatic HE between Sitagliptin and SU - HE complications
	India	27 June– 21 September, 2011	Summer	12.9 h per day							-
Arouj et al., 2013 (47)	Countries in the Middle East and Asia	Varied	Summer	Vildagliptin 3.41 ± 3.18) SU 4.45 ± 4.27)	Multicenter, post-authorization, prospective RCT	Vildagliptin (n = 669) (57.7% male) SU (n = 624) (59.8% male)	T2DM	≥ 18	≥ 12 months	Ramadan fasting Vildagliptin compared with SU	- HbA1c - The proportion of patients with ≥ 1 HE
Ahmedani et al., 2014 (48)	Pakistan	31 July– 29 August, 2011	Summer	12.99	Prospective, pre and post observational	682 (47.7)	T1DM & T2DM	T1DM (24.50 ± 9.80) T2DM (53.24 ± 10.73)	T1DM 8.53 ± 6.56 T2DM 8.85 ± 6.84	fasting	- symptomatic HE
AlKhaldi et al., 2019 (49)	Saudi Arabia (Abha)	16 May– 14 June, 2018	Summer	13.30	Cross-sectional observational	378 (51)	T1DM & T2DM	45	12	T1DM patients used to act rapidly and long-acting insulin (65%), while in T2DM, more than one third (38%) used OHA 8% were on insulin alone	- Incidence of HE and its risk factors
Azar et al., 2016 (50)	Algeria, India, Lebanon, Malaysia, South Africa, UAE, Israel	Varied	Summer	Varied	Randomized, parallel, open-label, active-controlled trial RCT	343 (49)	T2DM	Liraglutide 54.9 ± 9.27 SU 54.0 ± 9.33	Liraglutide 8.0 ± 5.26 SU 7.2 ± 4.39	Liraglutide SU	- Fructosamine levels - Symptomatic HE - HbA1c - FPG
Babineaux et al., 2015 (6)	India, Indonesia, Malaysia, France, Germany, UK, Turkey, Saudi Arabia, Kuwait, UAE, Algeria, Morocco, Tunisia	Varied	Summer	Varied	Multi-country, retrospective, observational	3,250 (48.5)	T2DM	56.9 ± 10.7	8.4 ± 6.3	OHAs (Oral only Monotherapy, Two-drug combination or Three-drug combination), Injectable only or Oral + injectable)	- FBG, PBG - HbA1c, - TC, TG, HDL, LDL
Ba-Essa et al., 2019 (51)	Saudi Arabia (Dammam)	05 May – 03 June, 2019	Summer	13.28	A prospective, non-randomized study for 2 years observational	360 (45.3)	T2DM	53.8 ± 9.3	12.5 ± 8.3	9 different types of medication patterns	- HbA1c - Incidence of HE
Bakiner et al., 2009 (52)	Turkey (Ankara)	01 September– 29 September, 2008	Summer	12.4	Prospective RCT	14 (57)	T2DM	50.14 ± 9.68	NR	Ramadan fasting	- FBG, PBG, - Fructosamine
Bashier et al., 2018 (53)	UAE (Dubai)	06 June– 05 July, 2016	Summer	15	Prospective trial RCT	535 (41.5)	T2DM	54.0 ± 11.6	13.4 ± 6.6	Ramadan fasting, SGLT2-I	- FBG, PBG, - HE
Bashier et al., 2019 (54)	UAE (Dubai)	16 May– 14 June, 2018	Summer	13.32	Prospective interventional trial RCT	67 (50)	insulin-treated patients with T2DM	55.7 ± 9.6	NR	Ramadan fasting	- FBG, PBG, - HbA1c - Lipid profile - frequency, duration, and severity of HE
Beshyah et al., 2019 (55)	Pakistan India, UAE, Kuwait, Saudi Arabia, Egypt	Varied	Summer	Varied	Cross-sectional multi-country observational	1795 (55.8)	majority with T2DM	51.3 ± 11.5	8.4 ± 6.8	Ramadan fasting	- Risk of HE and its impact on behavior
Bonakdaran, 2011 (56)	Iran (Mashhad)	01 September– 30 September, 2018	Autumn	12.3	Pilot, observational	17 (41)	T2DM	42.4 ± 8.5	6.9 ± 4.7	Only MTF or combination of MTF and Glibenclamide	- HbA1c - Effects of fasting on glycemic excursions by continuous glucose monitoring system (CGMS).
Brady et al., 2014 (57)	UK (Leicester and Birmingham)	2 rounds of Ramadan 2011 and 2012	Summer	Varied	RCT	99 (50)	T2DM	52	NR	Liraglutide (1.2 mg/day) or SU, in addition to MTF	- Lipid parameters - HbA1c - Weight - HE
Dabbous et al., 2019 (58)	Qatar	26 May– 24 June, 2017	Summer	14.29	Prospective study Pre and post Observational	181 (63.5)	T2DM	53.6 ± 9.7	10.6 ± 6.5	Ramadan fasting	- HbA1c - Lipid profile
Devendra et al., 2009 (59)	UK (North West London)	22 August– 21 September, 2009	Autumn	13.13	Observational before and after	Vildagliptin n=26 (61.5) Gliclazide n=26 (69.2) Total =52 (male 65.4)	T2DM	Vildagliptin 53.2 ± 9.7 (33–73) Gliclazide 62.3 ± 9.8 (45–77)	Vildagliptin 7.1 ± 3.1 (3.3–16.5) Gliclazide 6.9 ± 1.4 (4.5–11.2)	whose HbA1c was > 8.5% despite treatment with MTF 2 g daily before Ramadan and who received Gliclazide 160 mg twice daily (n = 26) or Vildagliptin 50 mg twice daily (n = 26) in addition to MTF	- HE - HbA1c - Weight
Eissa et al., 2017 (60)	Egypt (Sohag)	27 May–24 June, 2016	Summer	14.24	Prospective observational	90 (38.9)	T2DM	59.93 ± 7.87	4.52 ± 2.45 (1–10).	Group I: Patients using MTF alone Group II: Patients using SU with or without MTF Group III: Patients using Dipeptidyl peptidase-4 inhibitors Group V: Patients using Thiazolidinediones with or without MTF Group VI: Patients using combinations of more than two drugs	- HbA1c - Symptomatic HE, hyperglycemia - Weight
Elmehdawi et al., 2010 (61)	Libya (Benghazi)	21 August– 20, September, 2009	Summer	12.61	Cross-sectional observational	493 (50.3)	T2DM (about 95%) nor reported in T1 tables	59 ± 11.7	11.3 ± 10	Insulin alone or oral antidiabetic agents or diet alone	- HE
M’Guil et al., 2008 (8)	Morocco (Rabat)	December, 2001–January, 2002	Winter	8.88	Prospective observational	120 (48.3)	T2DM	48–60	4.5–5.5	Oral hypoglycemic drug	- FBG, PBG, - HbA1c - Lipid parameters
Halimi et al., 2013 (62)	France	20 July– 19 August, 2012	Summer	14.01	Prospective, non-interventional Study	218 (60)	T2DM	59	7.4 ± 5.2	MTF	- Rate of HE
Hassanein et al., 2011 (63)	UK	31 July– 29 August, 2011	Summer	14.37	Post-authorization, prospective, observational, non-interventional two-cohort	72 (55.9%)	T2DM	Vildagliptin 58.3 ± 13.1 SU 57.3 ± 11.0	Vildagliptin 7.1 ± 6.1SU5.8 ± 4.7	Vildagliptin (50 mg twice daily) or SU as an add-on to MTF	- Symptomatic & severe HE & % occurrence - HbA1c
Hassanein et al., 2014 (64)	16 countries across the Middle East, Europe, and Asia.	Varied	Varied	Varied	Multicenter, double-blind, double-dummy, randomized, active-controlled, parallel-group interventional clinical trial	557Vildagliptin N=132 (47.3)Gliclazide N= 128 (46)	T2DM	Vildagliptin54.3 ± 9.1Gliclazide 54.6 ± 9.3	Vildagliptin4.8 ± 4.1Gliclazide4.7 ± 3.8	- Ramadan fasting - Previously treated with MTF and any SU to receive either Vildagliptin (50 mg twice daily) or Gliclazide plus MTF	- HE
Hassanein et al., 2017 (65)	Lebanon, Kuwait, UAE	06 June– 05 July, 2016	Summer	15	Non-randomized, parallel-cohort, prospective, comparative, observational	SU n=159 CANA n=162 (58.3%)	T2DM	SU 54.3 ± 7.4 CANA 52.3 ± 7.7	SU 7.6 ± 5.5 CANA 6.5 ± 5.9	Canagliflozin or any SU added to MTF ± dipeptidyl peptidase-4 Inhibitor	- Symptomatic HE
Hassanein et al., 2018 (66)	Across 27 sites in Algeria, India, Lebanon, Malaysia, and South Africa	06 June– 05 July, 2016	Summer	12–16	Open-label, randomized, treat-to-target clinical the trial was conducted before, during, and after Ramadan RCT	471 (48.9)	T2DM	IDegAsp BID 54.9 ± 9.8 BIAsp 30 BID 55.3 ± 9.2	IDegAsp BID 12.1 ± 6.8 BIAsp 30 BID 12.3 ± 7.4	To compare the efficacy and safety of insulin degludec/insulin aspart (IDegAsp) and biphasic insulin aspart 30 (BIAsp 30) before, during, and after Ramadan	- HE - Glycemic control - HbA1c
Hassanein et al., 2019 (67)	Egypt, Iraq, Jordan, Saudi Arabia, Kuwait, Lebanon, Morocco, Pakistan, UAE, Israel	06 June– 05 July, 2016	Summer	Varied	Prospective, observational	1749 (55.6)	T2DM	55.2 ± 11.1	10.2 ± 8.0	- Oral antidiabetic drugs - Fasting	- HbA1c, - FPG, and PPG - HE - Lipid profile
Hui et al., 2010 (68)	UK (London boroughs of Brent, Ealing, and Harrow)	21 August– 20 September, 2009	Summer	13.17	Observational	Mix 50 N=26 (30.8) Mix 30 N=26 (38.5)	T2DM	Mix 50 62.3 ± 9.8 (45–77) Mix 30 61.9 ± 9.2 (44–77)	Mix 50 9.8 ± 2.8 (4.6–16.5) Mix 30 9.5 ± 3.3 (4.5–16.5)	Humalog Mix 50 and human Mixtard 30 twice daily during Ramadan fasting	- HE - HbA1c - Body weight
Jabbar et al., 2017 (69)	India, Indonesia, Malaysia, France, Germany, United Kingdom, Turkey, Saudi Arabia, Kuwait, UAE, Algeria, Morocco, Tunisia	Varied	Varied	Varied	Multi-country, retrospective, observational	1568 (48.5)	T2DM	56.9 (10.7)	8.4 ± 6.3	OHA	- Incidence of HE
Jamoussi et al., 2017 (70)	Tunisia (Tunis)	09 July– 07 August, 2013	Summer	13.91	Prospective RCT	54 (52)	T2DM	Educated group 53 ± 5.3 Non educated group 55 ± 8.6	Educated group 4.27 ± 4 Non educated group 6 ± 3.7	Education protocol	- HE - HbA1c - Lipid profile - Anthropometric
Khaled et al., 2009 (22)	Algeria (Sidi-Bel-abbès)	15 October– 14 November, 2004	Autumn	10.68	Cross-sectional observational	276 (0)	T2DM	49 ± 6	4 ± 2	Women receiving OHAs treated with MTF and/or Glimepiride	- Anthropometric - Food intake - HE
Khalifa et al., 2015 (71)	UAE (Dubai)	28 June– 28 July, 2014	Summer	13.32	Prospective observational Trial	111 (31.5)	T2DM	52.6 ± 10.1	12.2 ± 5	Liraglutide as an add-on therapy to existing OHA	- HE - HbA1c
Khatib et al., 2004 (72)	Jordan (Amman)	26 October– 26 November, 2003	Autumn	10.62	Pre and post observational	44 (100)	T2DM	52 ± 9 (35–75).	8.37 ± 7.02 (1–27)	Ramadan fasting	- FBS - HbA1c - Lipid profile - Body weight
Khattab et al., 2016 (73)	Egypt (Alexandria)	06 June– 05 July, 2016	Summer	14.06	Multicenter prospective and non-interventional	246 (55.7)	T2DM	49.0 (8.2)	3.0 ± 2.4	Vildagliptin or SU	- HE
Lum et al., 2020 (74)	Singapore	2 Ramadan cycles in 2017 and 2018	Summer	13.5	A prospective, multicenter, open-label, parallel-group, RCT	97 (40.2)	T2DM	59.5 (11.2)	10 (5.0–20.0)	Two groups (OHAs; OHAs and insulin	- FBG, PBG - Diabetes Stress - HbA1c
Mafauzy, 2002 (75)	Malaysia, UK, France, Saudi Arabia, Morocco	Varied	Varied	Varied	Open-label, multicenter, randomized, parallel-group RCT	235 (71.9)	T2DM	Repaglinide 52.7 (7.4) Glibenclamide 54.5 (6.9)	Repaglinide 7.2 ± 4.5 Glibenclamide 7.3 ± 5.0	Comparison between Repaglinide and Glibenclamide treatment	- Serum Fructosamine - HE - Midday BG
Malek et al., 2019 (76)	Algeria	26 May– 24 June, 2017	Summer	14.26	Prospective Observational	901 (41.8)	T1DM & T2DM	56.99 ± 11.54 (17–88)	T2DM 7.87 ± 5.97 T1DM 9.09 ± 8.19	- Ramadan fasting - Education - Adjustment of SMBG and medications (oral drugs and insulin).	- Blood glucose - HbA1c
Malha et al., 2014 (77)	Lebanon (Beirut)	28 June 28 July, 2014	Summer	13.89	Interventional randomized open-label clinical trial	69 (NR)	T2DM	Vildagliptin group 57.0 ± 9.6 Control group 54.6 ± 9.2	Vildagliptin group 9.8 ± 9.2 Control group 8.4 ± 6.4	Given Vildagliptin 50 mg twice daily compared to usual usage of MTF and SU regimen (glimepiride or gliclazide)	- HE - HbA1c - FBG
McEwen et al., 2018 (9)	Egypt, Iran, Jordan, Saudi Arabia	May–June 2014	Summer	Varied	Observational	774 (41e)	T2DM	48 ± 10	9 ± 5	Ramadan fasting Education	- Awareness about HE - HE
Norouzy et al., 2012 (78)	Iran (Mashhad)	01September– 30 September, 2008	Autumn	12.03	A prospective cohort clinical trial Observational	103 (47.6)	T2DM	51.3 ± 10.6	NR	Ramadan fasting	- Lipid profile - HbA1c - FBG - Blood pressure - Weight and BMI - Anthropometric
Sahay et al., 2020 (79)	India, Kuwait, Lebanon, Turkey, Israel	May 27 to June 24–25, 2017	Summer	Varied	Randomized, multicenter, open-label, 12–22-week, 2-arm parallel-group clinical trial RCT	150 (42.67)	T2DM	Lixisenatide + BI (50.7 ± 9.3) SU + BI (52.8 ± 10.7)	Lixisenatide + BI (5.9 ± 3.6) SU + BI (5.9 ± 4.0)	Lixisenatide + BI Or SU + BI	-Symptomatic HE
Salti, 2009 (80)	Bangladesh, China, Egypt, Kuwait, Oman, UAE, India, Indonesia, Lebanon, Jordan, Malaysia, Morocco, Saudi Arabia, Tunisia	Varied	Varied	Varied	Open, descriptive, multi-center, prospective RCT	441 (51)	T2DM	54.5 ± 9.2 Range (46 – 64)	11.3 ± 7.1	Insulin glargine and glimepiride	- HE - Glycemic control - FBG - HbA1c
Sari et al., 2004 (81)	Turkey (Antalya)	15 October- 14 November, 2004	Autumn	10.64	RCT	52 (NR) Group 1 (diabetic diet, n=12) Group 2 (single dose SU, n=13) Group (Repaglinide 2x2 mg, n=27)	T2DM	Group1 59 ± 6 Group2 57 ± 5 Group3 58 ± 8	Group1 2.7 ± 2.1 Group2 2.9 ± 1.9 Group3 3.3 ± 2.3	SU (Glimepiride or Gliclazide)	- Body weight - HbA1c - Lipid levels - Fructosamine - Glucose - β-Hydroxybutyric acid
Shehadeh et al., 2015 (82)	Israel (Haifa)	31 July– 29 August, 2011	Summer	13.01	Open-label, prospective, controlled multicenter cluster non-inferiority randomized RCT	(245) Intervention 50 (39.4) Control 44 (37.9)	T2DM	Intervention 60.1 (8.9) Control 59.4 (10.1)	Intervention 13.4 (6.1) Control 12.0 (5.3)	Insulin Detemir (Levemir) and Biphasic insulin (Novo Mix 70)	- HbA1c - Lipid profile - Fructosamine - symptomatic HE
Shete et al., 2013 (7)	India	10 August– 09 September, 2010	Summer	12	multicenter, non-interventional prospective, open-label, observational	97 (NR)	T2DM	Vildagliptin group (n = 55) 51.0 ± 8.8 SU (n = 42) 50.9 ± 9.1	NR	SU (Glibenclamide or Gliclazide or Glimepiride or Glipizide)	- Symptomatic HE - Body weight - FBG and PBG
Siaw et al., 2014 (21)	Singapore	21 July– 18 August, 2012	Summer	12.95	Prospective RCT	153 (37.3)	T2DM	56.7 ± 9.1	13.2 ± 9.1	Ramadan fasting OHA or Insulin or both together	- HbA1c - HE
Sifri et al., 2011 (83)	. Egypt, Jordan, Lebanon, Saudi Arabia, UAE, Israel	17 June–19 November, 2010	Varied	Varied	Open-label RCT	1066 Sitagliptin (n = 507) (53) SU (n = 514) (50)	T2DM	Sitagliptin (55 ± 11) (24,94) SU (55 ± 10 (23, 87)	Sitagliptin (5.0), SU (6.0)	SU with or without MTF, compared to Sitagliptin	- Symptomatic HE
Toony et al., 2018 (84)	Egypt (Assiut)	May, 2016–July, 2017	Summer	???	A prospective interventional controlled design RCT	320 (Control, n=200) (27) (Intervention, n=120 (21.7)	T2DM	Control 49.52 ± 9.8 Intervention 51.08 ± 9.6	Control 7.15 ± 6.5 Intervention 8.64 ± 6.9	Ramadan fasting Educational program	- Symptomatic HE - FBG - HbA1c
Tourkmani et al., 2016 (85)	Saudi Arabia (Riyadh)	08 July- 07 August, 2013	Summer	13.22	A prospective nonrandomized interventional controlled RCT	262 (Intervention n=140) Control n=122 (37)	T2DM	Intervention55.12 ± 12.76 Control 56.06 ± 11.08	Intervention12.95 ± 8.39 Control 12.86 ± 7.61	Ramadan fasting Ramadan Focused Education Program (RFEP)	- HE - HbA1c - Blood pressure - Body weight and BMI - Lipid profile
Tourkmani et al., 2019 (86)	Saudi Arabia (Riyadh)	08 July– 07 August, 2013	Summer	13.22	Controlled, intervention-based study pre and post RCT	262 Intervention n=140 Control n=122(37)	T2DM	Intervention55.12 ± 12.76 Control 56.06 ± 11.08	Intervention12.95 ± 8.39 Control 12.86 ± 7.61	Ramadan fasting, Ramadan Focused Education Program (RFEP)	- HE scores - HbA1c - The dose of MTF, aspart, Glargine, Mixtard
Vasan et al., 2006 (87)	India (Vellore)	15 October– 14 November, 2004	Autumn	11.41	multicenter, double-blind RCT	86 Pioglitazone n=43 n=26(60.5) Placebo n=44 n=8(18.2%)	T2DM	Pioglitazone45 ± 9 Placebo 45 ± 9	pioglitazone8.0 ± 2 placebo 8.5 ± 2.2	Pioglitazone compared to placebo in addition to the existing OHA	- Fructosamine - HE
Vasan et al., 2012 (88)	Sweden (Stockholm)	31 July– 29 August, 2011	Summer	15.35	An original multicenter, randomized double-blind, placebo-controlled trial RCT	70 (69.5)	T2DM	45 ± 7	8 ± 2	Ramadan fasting	- Dietary habits - Incidence of HE - Glycemic control
Wan seman et al., 2016 (89)	Malaysia (Kuala Lumpur)	28 June– 28 July, 2014	Summer	12.17	randomized, open-label, two-arm parallel group RCT	Total = 110 Dapagliflozin+ MTF n=58 (60.3) SU+MTF, n=52 (59.6)	T2DM	Dapagliflozin+ MTF 53 (9.1) SU+MTF 56 (9.1)	Dapagliflozin+ MTF 5.0 (3.0, 9.0) SU+MTF, 6.0 (3.0, 10.3)	Dapagliflozin compared with SU	- HE
Zargar et al., 2010 (90)	Bangladesh, Pakistan, India	Varied	Varied	Varied	Pre and post Observational	136 (100%)	T2DM	Bangladesh 51.1 ± 8.9 Pakistan 53.6 ± 11.1 India 50.2 ± 10.7	NR	Ramadan fasting	- FBG - HbA1c - Lipid profile - Body weight and BMI
Susilparat et al., 2014 (91)	Thailand (Pathumthani)	28 June– 28 July, 2014	Summer	12.52	Quasi-Experimental RCT	Experimental n=62 (30.65) Control group n=28 (17.86)	T2DM	Experimental group 56.26+9.481 Control group 54.79+9.85	NR	Education before Ramadan fasting and adjustment of anti-diabetic medicine	- Blood pressure - Incidence of HE - Weight and BMI - Anthropometric - FBG
Belkhadir et al., 1993 (92)	Morocco (Casablanca, Rabat	06 March– 05 April, 1992	Autumn	11.7	Non-randomized control	Total of 591 G1—Control n=194 G2—a full dose of Glibenclamide group, n=199 G3—a reduced dose of Glibenclamide group, n=198	T2DM	G1 57.2 (9.0) G2 54.9 (9.3) G3 54.8 (9.7)	Fasting + G18-53 (6-2) G2 7-03 (5-5) G3 7-05 (5-35)	Ramadan fasting + Glibenclamide	- Fructosamine - HbA1c - Body weight
Lessan et al., 2015 (93)	UAE (Abu Dhabi)	28 June– 28 July, 2014	Summer	14.20	Cross-sectional observational	63 (61.9)	T2DM	44.9 ± 12.1	NR	Ramadan fasting + G1 = diet with/without MTF G2= gliptin with/without MTF G3= SU with/without other oral agent(s) G4= insulin with/without other oral agents	- HbA1c - FBG - Incidence of HE
Almalki et al., 2018 (13)	Saudi Arabia (Riyadh)	16 May– 14 June, 2018	Summer	13.30	Cross-sectional RCT	477 (31.9)	T1DM & T2DM	39.72 ± 15.29	10.80 ± 5.88	Diabetes treatment & education	- Symptomatic HE
Malik et al., 2017 (94)	Pakistan (Lahore)	06 June– 05 July, 2016	Summer	14.08	Cross-sectional Observational	33 (57.6)	T2DM	51.15 ± 9.36	NR	Blood glucose monitoring during Ramadan fasting & education	- Symptomatic and biochemical hypoglycemia

*NR, Not reported.

CGMS, Continuous glucose monitor; FBG, Fasting blood glucose; FPG, Fasting plasma glucose; GMR, Gliclazide modified release; HE, Hypoglycemic event/episode; MTF, Metformin; OHA, Oral hypoglycemic agent; RCT, Randomized-controlled trial; SGLT2i, Sodium/glucose cotransporter-2 inhibitors; SU, Sulfonylureas; UAE, United Arab Emirates.

Out of the 68 selected papers, there were 35 randomized controlled trials (RCTs) and 33 observational studies. Because of the nature of Ramadan month and its connection with the lunar calendar, Ramadan fell in different solar seasons in the included studies. The majority of the studies were conducted during the summer season, especially those conducted during 2010–2018. There was wide variation in the age of study participants, ranging from 17 to 94 years. Also, there was variation in the duration of fasting (9–17 h/day) associated with different geographical locations and seasons. Participants in the included studies were examined for HE and cardiometabolic risk factor outcomes.

Patients with diabetes in the included studies used different types of medications during the fasting month. This included insulin-treated patients with T2DM that used an insulin pump, multiple daily injections, insulin lispro, insulin glargine, soluble human insulin, insulin detemir (Levemir), and biphasic insulin. Different types of OHGAs were also reported, such as biguanides (MTF), SU (gliclazide, glipizide, glimepiride, glibenclamide, or glyburide), DPP-4 inhibitors (sitagliptin, vildagliptin), thiazolidinediones (pioglitazone), SGLT2-I (dapagliflozin, canagliflozin), incretin mimetics, and GLP-1 receptor agonists (lixisenatide injection, liraglutide).

The included studies were conducted across 33 countries in four continents. This included six African countries: Algeria (n=6), Egypt (n=14), Libya (n=1), Morocco (n=8), South Africa (n=3), and Tunisia (n=5). There were also studies from 19 Asian and Middle Eastern countries: Bangladesh (n=4), India (n=17), Indonesia (n=6), Malaysia (n=13), Pakistan (n=11), Singapore (n=4), Thailand (n=1), China (n=1), Iran (n=3), Iraq (n=1), Jordan (n=7), Kuwait (n=12), Lebanon (n=12), Oman (n=2), Qatar (n=3), Saudi Arabia (n=19), Turkey (n=9), Israel (n=7), and the United Arab Emirates (UAE) (n=20). There were studies from seven countries in Europe: France (n=4), Germany (n=3), Sweden (n=1), the UK (n=8), Spain (n=1), Russia (n=1), and Denmark (n=1). Finally, one study from North America was located (Canada: n=1). Eleven countries that were represented in this review were from the Middle East region (the UAE, Saudi Arabia, Jordan, Lebanon, Kuwait, Qatar, Oman, Turkey, Israel, Iraq, and Iran); these countries include about one-fifth of the global Muslim population.

In total, there were 26,706 participants in the included studies, of which 48.6% were male (n=12,988). Three studies (n=218 participants) did not mention the percentage of males in the study population, which was a major drawback in those studies. Most studies were conducted in places where Ramadan fell in summer (n=50). Nine studies were conducted in autumn, one in winter, and one in spring. Besides, seven studies considered participants in countries across different continents; therefore, they reported varied seasons. The minimum fasting duration (hours) was 8.88 h, and the maximum was 16.5 h, with average fasting hours of 13.22 h. The youngest mean age of participants was 24.5 years, and the oldest mean age was 62.1 years (age range: 17–94 years). Similarly, the shortest mean duration since diabetes diagnosis was 2.97 years, and the longest was 15 years (range: 0.3–32.9 years). **Table 2** summarizes the HE outcomes reported in the experimental studies (RCTs) involving patients with T2DM observed during Ramadan fasting month. HE reported in the observational studies involving patients with T2DM observed during Ramadan fasting month are summarized in **Table 3**.

**Table 2:** Description of the experimental studies regarding hypoglycemia and hypoglycemic events during Ramadan fasting among patients with T2DM.

Study authors	Type of HE*	Reference value used in diagnosing hypoglycemia	Hypoglycemia description
Ahmedani and Ghafoor (18)	Recorded, severe	<70 mg/dl	Only one episode of major HE was reported in one of the patients immediately before *Iftar*. None of the patients developed diabetic ketoacidosis or hyperosmolar hypoglycemic state, and no one needed hospitalization. Only one episode of major hypoglycemia was reported in one of the patients immediately before *Iftar* (Pre-dusk meal).
Mattoo et al. (38)	Recorded, symptomatic	<63 mg/dl	The mean number of HE per patient per 14 days was similar in the two groups of Lispro Mix25 and insulin. Both regimens were well-tolerated and did not increase the risk of HE.
Hassanein et al. (95)	Documented, symptomatic	<70 mg/dl	A numerically lower percentage of participants with Lixisenatide + basal insulin (BI) vs. SU+BI had >1 documented symptomatic HE during Ramadan fasting; the difference was statistically significant for the ‘any hypoglycemia’ category. Compared with SU + BI, lixisenatide + BI provided lower rates of any HE in people with T2DM during Ramadan fasting.
Akram et al. (41)	Recorded	< 3.5 mmol/L	No severe cases of HE were reported. Mean HE per patient over 14 days was significantly lower in insulin Lispro than soluble insulin. Most HE occurred during the time from 6 h after the before sunrise meal until breaking the fast after sunset. Insulin Lispro may be more suitable prandial insulin for patients treated with T2DM who fast during Ramadan.
Anwar et al. (42)	Recorded, symptomatic	<3.1 mmol/L	18 HE were recorded, six events by repaglinide-treated patients and 12 events by glimepiride-treated patients, without statistical difference between the two groups during and after Ramadan.
Abdelgadir et al. (43)	Symptomatic	NR	Both the severity and duration of HE reduced during the month of Ramadan. The SGLT2i group had shorter and less severe episodes in comparison to the non-SGLT2i group.
Aravind et al. (32)	Recorded, symptomatic	≤ 70 mg/dl	Symptomatic HE was reported during Ramadan by patients who were lower in the sitagliptin group than the SU group. The number of patients reporting at least two symptomatic HE was three in the sitagliptin group and nine in the SU. The proportion of patients with symptomatic HE confirmed with a corresponding BG≤ 70 mg/dl was 2.1% in the sitagliptin group and 5.4%in the SU group. One patient (0.2%) in the sitagliptin group and two (0.5%) in the SU group reported symptomatic HE that had a corresponding BG <50 mg/dl.
Arouj et al. (47)	Recorded, symptomatic	< 3.9 mmol/l (70 mg/dl)	Fewer patients experienced HE with vildagliptin in comparison to those who received SU. The majority of patients who experienced HE during fasting reported a lower number of single HE in the vildagliptin group than in the SU group. A smaller proportion of patients experienced 1 HE with vildagliptin when compared with commonly used individual SU, with the highest proportions of HE in the glibenclamide group, followed by gliclazide, glimepiride, and glipizide.
Azar et al. (50)	Documented, symptomatic	<70 mg/dl	Fewer reported symptomatic HE was reported in liraglutide-treated than SU, with no severe HE was reported by either medication group.
Bakiner et al. (52)	Recorded	<60 mg/dl	The participants reported neither a major nor a minor HE.
Bashier et al. (53)	Recorded, symptomatic	<70 mg/dl	Confirmed HE was reported in the vast majority of the study sample. The HE was significantly more frequent in the SGLT2-I plus insulin-treated group than in those treated with SGLT2-I plus OHGA group. Confirmed HE was more frequent in those using SGLT2-I plus intensive insulin compared to those using SGLT2-I plus basal insulin.
Bashier et al. (54)	Recorded, symptomatic	<70 mg/dl	The average number of HE numerically, not significantly, reduced before Ramadan to during Ramadan. As well, the duration of HE was reduced numerically during Ramadan.
Brady et al. (57)	Recorded	≤3.9 mmol/l	No severe HE in either group of MTF + liraglutide and MTF+SU. More participants in the SU experienced one or more HE during the study compared within the liraglutide group, with a median of three events per participant in the SU group compared with two in the liraglutide group. The incidence rate of HE was 10.5 per person-year in the SU group compared with 3.0 per person-year in the liraglutide group.
Hassanein et al. (63)	Recorded, severe	<3.9 mmol/L	No HE or severe HEs with MTF + vildagliptin, while HE and one severe HE were reported. While vildagliptin was very well tolerated, with no Adverse Events (AE), severe AEs, or deaths reported half of the patients on SU reported at least one AE. The main driver for this difference was the high incidence of hypoglycemia.
Hassanein et al. (64)	Recorded, symptomatic	<3.9 mmol/L	The proportion of patients who reported any HE during the Ramadan fasting period was numerical, not significantly, lower in the MTF+ vildagliptin compared with the MTF + gliclazide. Fewer patients in the vildagliptin group developed HE than patients in the gliclazide group. There were no severe HEs reported in either treatment group.
Hassanein et al. (66)	Recorded, symptomatic, severe	< 3.1 mmol/L	The rate of overall HE was lower in the insulin degludec/insulin aspart (IDegAsp) arm compared with the biphasic insulin aspart 30 (BIAsp 30) arm, with a 74% reduction in the rate of overall HE. The rate of nocturnal HE was also lower in the IDegAsp arm compared with the BIAsp 30 arm, translated into more than 80% reduction in the rate of nocturnal HE in patients receiving IDegAsp. The rate of severe HE was 44% lower, in the IDegAsp arm compared within the BIAsp 30 arm. During Ramadan, a lower rate of overall HE (more than 60% reduction) was observed in the IDegAsp arm compared with the BIAsp 30 arm. DegAsp arm had a lower rate of nocturnal HE and daytime HE compared with those in the BIAsp 30 arm achieving 74 and 56% reductions in rates of nocturnal and daytime HE, respectively
Jamoussi et al. (70)	Recorded	<0.7 g/l	One patient from each group presented HE is requiring breaking the fast, no severe HE was reported. The group that received diabetes education before Ramadan showed a lower frequency of minor HE before and during Ramadan than the control group
Khattab et al. (73)	Recorded, symptomatic	<3.9 mmol/L	A lower proportion of patients experienced HE in the vildagliptin group than in the SU group during Ramadan. No patient in either group experienced a severe HE during Ramadan. None of the HEs led to discontinuation from the study
Lum et al. (74)	Symptomatic, recorded	<72 mg/dl	No self-reported major HE was experienced during Ramadan for control and intervention groups. A total of 30.4% and 29.4% incidents of self-reported minor HE during Ramadan were reported in the intervention and control group, respectively. Only 2.2% in the intervention group and about 10% in the control group were actual HE confirmed by SMBG.
Mafauzy (75)	Symptomatic, recorded	<2.8 mmol/l (HE) or <2.8 mmol/l (hypoglycemic symptoms)	HE recorded in patients’ diaries were categorized into those with HE or < 2.8 mmol/l with hypoglycemic symptoms. During Ramadan, 7% on repaglinide-treated patients compared with 8% glibenclamide-treated patients reported HE. Fewer repaglinide-treated patients had HE compared with glibenclamide treated patients.
Malha et al. (77)	Symptomatic, recorded	<70 mg/dl	HE was numerically higher in the SU group than the vildagliptin group. The number of patients experiencing HE was higher in the SU group compared with the vildagliptin group. Patients breaking the fast due to intolerance to fasting, discomfort, and fear of HE were all in the SU group, and there were no patients in the vildagliptin group who discontinued their fasting.
Sahay et al. (79)	Documented, symptomatic	<70 mg/dl	Less participants on lixisenatide + basal insulin (BI) experienced > _1 documented symptomatic HE during Ramadan than participants on SU + BI(1.3% vs. 6.8%, respectively)e. Incidence of any HE was numerically lower with lixisenatide + BI vs. SU + BI during Ramadan(1.3% vs. 14.7%, respectively).
Salti (80),	Symptomatic	<3.3 mmol/	Mild HE significantly increased from pre-Ramadan to during Ramadan and significantly decreased to post-Ramadan. The increase during Ramadan was mainly attributed to increased symptomatic HE.
Sari et al. (81)	Recorded, symptomatic	40 mg/dl	Only one HE occurred on the 6th day of Ramadan in patients on 3 mg glimepiride.
Shehadeh et al. (82)	Symptomatic	<70 mg/dl	HE rate was significantly lower in the intervention group (insulin dose) than the control group on standard care. Treatment with Levemir and Novo Mix 70 was non-inferior to standard care in this heterogeneous group of patients and was associated with fewer adverse events.
Siaw et al. (21)	Symptomatic	NR	About a 25% rate of minor HE occurred in fasting patients with diabetes. While any form of HE is undesirable in the management of diabetes, this rate is nevertheless lower than or comparable with other studies conducted in non-fasting patients with T2DM.
Sifri et al. (83)	Symptomatic	<70 mg/ dl (3.9 mmol/l)	The risk of symptomatic HE was significantly decreased with Sitagliptin relative to SU. No reported severe HE or that required medical assistance was reported during Ramadan.
Toony et al. (84)	Symptomatic	Hypoglycemia was classified	HE during fasting occurred in patients in the intervention group (individualized diabetic education sessions before Ramadan.) than in the control group on standard care. (4.1% vs. 19.5%, respectively.
Tourkmani et al. (85)	HE scores		The hypoglycemic scores before, during, and after Ramadan was lower in the intervention (pre-diabetes education with an antidiabetic agent) group in comparison with the control (standard care) group.
Tourkmani et al. (86)	HE scores	NR	The hypoglycemia score in the intervention (Ramadan structured education) group was reduced from pre-Ramadan into during Ramadan while in the control (standard care) group, no significant changes were noted before and during Ramadan.
Vasan et al. (87)	Self-reported	NR	No significant difference in the number of self-reported HE between the two groups (Pioglitazone and placebo). No reduction in the number of reported HE when compared with conventional therapy without pioglitazone.
Vasan et al. (88)	Self-report	NR	Hypoglycemia assessment (based on self-reports) was not seen among study participants similar to previous observations in fasting diabetic patients treated with anti-diabetic drugs or insulin. They did not see any hypoglycemic events because of a sufficient energy diet pattern.
Wan seman et al. (89)	Reported or documented	<3.9mmol/l	A lower proportion of patients had reported or documented HE in the Dapagliflozin group than in the SU group. The relative risk of any reported or documented HE in the 4th week of Ramadan was significantly lower in the Dapagliflozin group.
Susilparat et al. (91)	Severe, symptomatic	NR	There was no incidence of severe HE in either experimental or the control group. The number and portion of patients with HE symptoms in the experimental group were lower than those in the controlled group
Almalki et al. (13)	Symptomatic, self-reported	NR	HE were reported in 60.3% of cases with T2DM and in 8.3% of cases with T1DM. Among those who had hypoglycemia, 2.8% of patients with T1DM and 17.8% with T2DM broke their fast.

*Type of hypoglycemic event means: recorded, symptomatic, or severe. If the study did not compare between pre and post and compare two types of medication, this will be described in the “post-Ramadan” column.

**Table 3:** Description of the observational studies regarding hypoglycemia and hypoglycemic events during Ramadan fasting among patients with T2DM.

Study authors	Type of HE*	Reference value and unit used in diagnosing HE	HE description
Cesur et al. (37)	Recorded, symptomatic	<70 mg/dl	At least one reported HE was found in 12.2% of patients in the fasting group. HE was observed higher in the glimepiride group, less in the repaglinide group, and least in the insulin glargine group. There was no significant difference between the three-drug groups regarding the rate of HE.
Bashir et al. (39)	Symptomatic, documented HE	Clinical HE (presence of symptoms of hypoglycemia + capillary blood glucose of <4.0 mmol/l), CGMS hypoglycemia (glucose <3.9 mmol/l or 70 mg/dl)	HE is quite frequent in patients with T2DM who were on three or more anti-diabetic agents during Ramadan
Hassanein et al. (34)	Recorded, symptomatic	<70 mg/dl	Patients with T2DM treated with Gliclazide MR during Ramadan have a low risk of HE and maintain glycemic control and weight while fasting. Patients with T2DM treated with Gliclazide MR without dose adjustment during Ramadan can fast safely with a low risk of HE and no risk of severe hypoglycemia, even in a prolonged fasting period, whilst maintaining glycemic control and weight.
Aldawi et al. (44)	Symptomatic	<70 mg/dl	One patient had who was on MTF only showed symptomatic HE during the early-Ramadan. T No serious HE was reported by the patients during Ramadan fasting, and none of the patients broke her/his Ramadan fasting. No significant differences were seen in the percentage of HE during early- and late-Ramadan compared to pre-and post-Ramadan.
Hassanein et al. (45)	Symptomatic	SMPG ≤70 mg/dl	The number of participants experiencing ≥1 event of severe and/or symptomatic documented HE was low and similar in the pre-Ramadan and Ramadan periods and was even lower in the post-Ramadan period. No patients reported severe HE during Ramadan and post-Ramadan periods; a single participant (0.2%) reported severe HE before Ramadan. During Ramadan, 11 out of the 13 participants who reported symptomatic documented hypoglycemia experienced HE during fasting hours. The proportion of participants experiencing a daytime vs nocturnal severe and/or symptomatic HE before Ramadan was two times higher during day hours than night hours. The numerical event rates (per participant-month of follow up [PPM]) for severe and/or symptomatic documented HE was higher during Ramadan than pre and post-Ramadan.
Aravind et al. (46)	Recorded, symptomatic, severe	≤70 mg/dl [3.9 mmol/L	About one-fifth of the patients reported one or more symptomatic HE during Ramadan. 207 out of 271 patients were reported HE reported a change in their anti-hyperglycemic medication during Ramadan, with headache, sweating, tremor, and palpitations were the most commonly reported symptoms. By treatment, the highest incidence of symptomatic HE was observed in subjects treated with glipizide or glibenclamide, followed by those treated with glimepiride or gliclazide. The average time between the last meal and the start of the HE was 8.1 ± 3.8 h for the entire cohort with no marked differences among SU groups. The incidence of documented HE was 3.6% overall and generally similar across SU groups. The highest incidence of severe types of HE occurring in the glibenclamide group. No marked differences were observed across SU groups. By study end, only 1.2%of all subjects experienced serious complications related to HE during Ramadan.
Ahmedani et al. (48)	Symptomatic, measured	<70 mg/dl	Overall, symptomatic HE was observed in 92 (23.7%) patients; in about 23% of patients with T2DM. About 71% of patients with T2DM checked Blood glucose levels when they developed HE during Ramadan. On the development of HE, 48% of patients with T2DM discontinued their fasting. Overall, HE was felt by 16.30% of patients; in 15.4% of patients with T2DM.
Alkhaldi et al. (49)	Symptomatic, recorded	<70 mg/dl	This study revealed that the incidence of hypoglycemia among diabetics was high More than half of the patients reported at least one HE during Ramadan, 29% out of them had more than four HE sacks. More than one-third of patients (39%) detected HE by symptoms, while less than half had symptoms and confirmed HE by glucometer. Less than one fourth have hypoglycemia at night, while about two-thirds of attacks (67%) occurred in the morning and evening while
Babineaux et al. (6)	Reported symptomatic	<70mg/dl	About 9% of patients reported at least one HE. Most of the HE required either assistance and/or stopping the fast. Hospitalization during Ramadan was rare.
Ba-Essa et al. (51)	Recorded, symptomatic	<70 mg/dl	The overall rate of hypoglycemia was about 25% with one episode of major HE requiring medical assistance. HE was significantly the highest in patients treated with insulin followed by those treated with oral agents including SU as compared to oral agents excluding SU. Those who experienced HE before Ramadan had the highest rate of HE during Ramadan.
Beshyah et al. (55)	Recorded, symptomatic	NR	About 16% of patients reported HE while the rest did not recall experiencing any hypoglycemia. Hypoglycemia was significantly associated with all insulin except basal insulin. It was also associated with Pioglitazone, SU, and MTF. Insulin in combo with any OAH drug was also associated with HE.
Bonakdaran et al. (56)	Recorded with or without symptoms	<3.88mmol/L	The extent and duration of HE was not significantly different between before and during Ramadan. The overall incidence of Asymptomatic HE was increased in Ramadan but not significantly. The patients reported neither a major nor a minor HE. A significantly increased number of HE in Ramadan was found in patients who take just SU or SU and MTF compared with those who take only MTF.
Devendra et al. (59)	Symptomatic, recorded	<3.5 mmol/L	Vildagliptin was associated with a reduction in the mean number of HE during Ramadan compared with before Ramadan (when patients were on MTF monotherapy), whereas gliclazide was associated with an increase. The number of patients experiencing HE was significantly lower in the vildagliptin group than in the gliclazide group. The total number of HE was 24 and 2 for the gliclazide and vildagliptin arms, respectively. There was one severe HE in the gliclazide group and none in the vildagliptin group.
Eissa et al. (60)	Symptomatic, recorded	<70 mg/dl	About 20% experienced one or more symptomatic HE during Ramadan, with incidences of about 26%, 17%, and 14.0% observed in subjects treated with glibenclamide, glimepiride, and gliclazide, respectively.
Elmehdawi et al. (61)	Reported, symptomatic, severe	NR	Mild HE was reported three times greater than severe HE, with no significant difference between patients with T1DM and T2DM regarding the number of days fasted, frequency of HE or severe HE, or admission rate during Ramadan. The incidence of hypoglycemia during Ramadan was numerically much higher than the incidence of severe hyperglycemia. About three-fourth of the fasting diabetic patients. The vast majority experienced hypoglycemia and about 80% experienced HE during the first 2 weeks of Ramadan, and about 90% of all HE occurred during the daytime. Females had a significantly higher frequency of severe HE than males. Any hypoglycemia was the most prevalent, followed by mild hypoglycemia, severe HE, and last hypoglycemia requiring admission.
M’Guil et al. (8)	Recorded	<0.50 g/L	No severe HE or hyperglycemia or hospitalization was reported by the patients. However, three episodes of HE with glucose levels of 0.49 g/L (a value lower than 0.50 g/L of glucose were considered as hypoglycemia) were noted right before breaking the fast (*Iftar*) in the same patient, but there were no accompanying symptoms.
Halimi et al. (62)	Recorded, symptomatic severe	≤70 mg/dl	Rates of HE were numerically lower in the MTF + vildagliptin group when compared with the MTF+ Insulin secretagogue (IS)/glinide. At least 1 episode of symptomatic HE was reported during Ramadan in 37.2% of patients in the IS group and 34.2% of patients in the vildagliptin group. The proportion of patients in whom at least 1 HE was declared confirmed by self-testing was numerically higher in the IS than in the vildagliptin group. Significantly more HE was documented by physicians as adverse events (AEs) in the IS group compared with the vildagliptin group. The proportion of patients reporting at least 1 severe HE that needs assistance was higher in the IS than in the vildagliptin group. Patients in the IS group were about six-times greater to have at least one unscheduled medical visit during Ramadan because of HE than the vildagliptin group.
Hassanein et al. (65)	Recorded, symptomatic	<3.9 mmol/L	Fewer patients on canagliflozin reported ≥1 symptomatic HE compared to patients treated on SU during Ramadan. The odds of having HE were lower in patients treated with canagliflozin.
Hassanein et al. (67)	Recorded, symptomatic, severe	<70 mg/dl	In the DAR-MENA T2DM population, the incidence of HE increased significantly from 4 weeks before versus during Ramadan for both confirmed hypoglycemia and severe hypoglycemia. Confirmed hypoglycemia AEs was significantly higher during Ramadan compared with 4 weeks before Ramadan, whereas the severe hypoglycemia AEs were comparable. The change is confirmed hypoglycemia, and corresponding AE, were dependent on the treatment regimen. People prescribed OADs without SU fasted with no statistically significant increase in confirmed hypoglycemia incidences or AEs from 4 weeks before Ramadan to during Ramadan while a significant increase occurred with those receiving OADs with SU There was no.
Hui et al. (68)	Symptomatic	< 3.5 mmol/l	Humalog Mix 50 and human Mixtard 30 twice daily during Ramadan fasting were tested on fasting patients with T2DM. Mix 50 was associated with a small reduction in the number HE during Ramadan compared with before Ramadan, whereas Mix 30 was associated with a non-significant increase.
Jabbar et al. (69)	Reported, symptomatic, severe	<70 mg/dl	The incidence of documented HE was greater in participants on insulin treatment than in participants on OHA alone, both before and during Ramadan. During Ramadan, about 19% of T2DMcohort treated with insulin alone had HE, compared with nearly 5% treated with OHA alone. About two-thirds of individuals receiving OHA alone were on SU. The incidence of HE during Ramadan with insulin treatment was highest in North Africa and lowest in Asia. T2DM treatment with pre-mixed insulin during Ramadan resulted in higher incidences of HE when compared with long-acting insulin. The incidence of HE during Ramadan for participants treated with a combination of OHA and insulin was similar for those treated with insulin alone. The incidence of HE in North Africa with OHA was significantly higher than in all other regions both before and during Ramadan. AHE before Ramadan increases an individual’s risk of having an episode during Ramadan. For individuals with T2DM who had no pre-Ramadan HE, 5% had an HE during Ramadan. For individuals who had ≥1 HE before Ramadan, 34.9% had ≥1 HE during Ramadan. This relationship between HE before and during Ramadan was consistent for all four regions.
Khaled et al. (22)	NR	NR	No, observe any frequent HE reported among diabetic patients treated with MTF and/or glimepiride, except for three cases: two patients took their medication without eating “Sahur” and one expended an important amount of physical energy.
Khalifa et al. (71)	Recorded and symptomatic	<70 mg/dl	About 16% of fasting patients developed HE during Ramadan without requiring medical assistance or admission.
Malek et al. (76)	Self-reported symptomatic	NR	HE was observed in about 20% and was more frequent in T2DM. The number of hypoglycemia reported in 135 patients, ranged at an average of 2.87 ± 2.33. About 62% HE was documented with an average of 59 ± 10 mg/dl. About 13% have severe HE led to the immediate break of the fasting.
McEwen et al. (9)	-Self-reported	NR	There were more mild and moderate HE reported by participants who received individualized education than those who received usual care, but fewer reported severe HE during Ramadan
Shete et al. (7)	Symptomatic	NR	HE was reported in low frequencies in both the Vildagliptin and the SU groups.
Zargar et al. (90)	Self-reported symptomatic	hypoglycemia (defined as any symptom of excessive hunger, perspiration, trembling, palpitation	HE reduced during and after Ramadan fasting in comparison with the pre-fasting T2DM male patients undertaking the Ramadan fast can safely maintain glycemic control with evening administration of Gliclazide MR 60 mg during the fast and reverting to a morning schedule thereafter. There were about 4% HE before Ramadan, about 2% during, and 1.5% after Ramadan.
Belkhadir et al. (92)	Symptomatic		No significant differences between the groups in the number of HE during Ramadan. In particular, no increase in HE was reported during fasting compared with before and after Ramadan.
Lessan et al. (93)	CGM recorded	Interstitial glucose ≤ 3.9 mmol/L	The overall HE rates were 1.11% during Ramadan and 2.41% during non-Ramadan periods; the difference was not statistically significant. No significant difference was found between fasting and non-fasting subjects concerning low blood glucose indices and the rate of HE.
Malik et al. (94)	Symptomatic, biochemical hypoglycemia		no extreme fluctuations in blood glucose levels only one patient reported symptomatic and biochemical hypoglycemia severe enough to break the fast at noon.

*Type of hypoglycemic event means: recorded, symptomatic, or severe. If the study did not compare between pre and post and compare two types of medication, this will be described in the “post-Ramadan” column.

## Discussion

The Ramadan and Diabetes Practical Guidelines (96) classifies people with diabetes that had severe hypoglycemia within the 3 months before Ramadan, a history of recurrent hypoglycemia, or a history of hypoglycemia unawareness as a very high-risk group. The guidelines, therefore, recommend that these people should not observe Ramadan fasting.

The patient’s awareness and understanding of the complication associated with fasting, including the possibility of developing HE, is highly important. Several reports have highlighted the positive effect of Ramadan-focused diabetes counseling and education for patients with diabetes before Ramadan on lowering the incidence and severity of HE during fasting (17, 84–86, 97, 98). A recent systematic review by Tourkmani and colleagues explored the impact of Ramadan-focused diabetes education on HE risk and metabolic control for patients with T2DM and highlighted the crucial impact of education and awareness (99). That review found a significant reduction in HE risk (by 81%) for fasting patients in intervention groups that received Ramadan-focused education compared with patients receiving conventional care. Furthermore, HbA1c was significantly improved among patients who received a Ramadan-focused diabetes education intervention compared with those receiving conventional care (99).

### Incidence of HE

The incidence of HE before Ramadan was associated with the incidence of HE during Ramadan among fasting patients with T2DM, as revealed in the CREED study (69). This was supported by another study that reported HE before Ramadan was associated with an increased risk for experiencing HE during Ramadan (51). Among individuals with one or more HE before Ramadan, 34.9% had one or more HE during Ramadan. This relationship between HE before and during Ramadan was consistent across all studies from four regions (Middle East, Europe, North Africa, and Asia). Another factor associated with HE among fasting patients with diabetes was the use of insulin therapy. Insulin therapy is associated with HE among patients with diabetes (100), and a significant correlation was found between the occurrence of HE during Ramadan and the use of insulin therapy before and during Ramadan (69).

### Biochemical Variations and HE

A key reason for the variability in reporting HE among patients with diabetes fasting during Ramadan was differences in the biochemical reference ranges used for a blood glucose level. For example, some studies used <3.1 mmol/L to assess hypoglycemia, others (i.e., the majority) used 3.9 mmol/L, and still, others used 4.4 mmol/L. Another reason for this heterogeneity was variation in defining HE. For example, some authors reported any HE, some reported only severe episodes, and others reported: “any hypoglycemia” (mild or severe) reported, documented, or biochemically measured among participants. Other possible reasons for the variability in reporting HE as the primary outcome across different studies included: variations in study design, intervention type (e.g., insulin titration, mixing different types of OHGAs, combining insulin or OHGA therapy with/without patient education about fasting), and type of dietary modifications and whether participants were adhering to healthy balanced diet during the feasting/eating night hours. Differences in the method of detecting hypoglycemia across the studies (e.g., using a diary, office visits, mobile application, or other methods) also contributed to this variation.

### Environmental Variations and HE

Variation in geographical locations and subsequent differences in the time and duration of fasting, climatic conditions, socioeconomic status, cultural/socio-cultural backgrounds, and dietary and lifestyle behaviors may all influence diabetic complications related to glycemic control during Ramadan, especially HE. For example, one study found that a lower proportion of Malaysian patients reported symptomatic HE compared with those from India (32). A multicenter study found the highest rate of symptomatic HE was reported among patients from Israel, followed by Malaysia, the UAE, India, and Saudi Arabia (46). Further, the CREED study reported patients with T2DM fasting during Ramadan that were resident in North Africa and Europe had higher odds of HE than those in the Middle East (69). That study also noted the overall incidence was significantly different (P < 0.0001) across regions, ranging from 4.1% in the Middle East to 13% in North Africa. This finding highlighted the effects of geographical location and dietary/cultural background in influencing glycemic control during Ramadan fasting. Further, the degree of strictness in practicing fasting by patients with diabetes varies among countries, which also affects the extent to which diabetic complications (including hypoglycemia) will occur. Salti and colleagues (80) found that the risk for HE during Ramadan was higher in Indonesia, Malaysia, Saudi Arabia than in Egypt, Morocco, and Tunisia.

### Dietary Patterns and Anthropometric Variations

It has been repeatedly reported that HE often occurred during the last 4–6 h before the end of fasting (43). SGLT2-I treatment showed a superior effect in lowering both the severity and duration of HE during Ramadan month, which may partly be explained by pre-Ramadan dose adjustment by treating physicians or patients’ self-titration after receiving Ramadan-focused education. The main reason behind these episodes was reported to be missing the pre-fasting pre-dawn meal (i.e., *suhoor*) while taking the prescribed dose of insulin (18).

Dietary intakes and eating behaviors differ among fasting people during Ramadan, especially in adherence to eating *suhoor*. This meal is important as it helps people with diabetes prevent anticipated hypoglycemia during fasting hours. This was reported among patients with diabetes in Malaysia, where eating *suhoor* reduced the risk for HE by more than two-fold compared with missing this meal (101). The length of fasting duration during Ramadan is another variable that impacted the occurrence of hypoglycemia and HE among fasting patients with diabetes during Ramadan. This is supported by repeated reports that most HE occurred in the daytime hours before *iftar* (sunset “breakfast” meal) among patients with diabetes observing Ramadan fasting (8, 45, 49, 51, 54, 61, 71, 102–104).

Vasan and colleagues (88) indicated that most fasting patients reported that consuming large quantities of food that yielded sufficient energy could help them sustain fasting and prevent hypoglycemia. Increased consumption of dietary fat, especially saturated fats, has been reported among Muslims during fasting periods. Although binge eating of high-calorie food is contrary to prophetic guidance and religious preaching, the main concern among fasting patients with diabetes was an interruption of continuous 30-day fasting due to hypoglycemia (88). Norouzy et al. highlighted the significance of dietary management in mitigating and ameliorating metabolic abnormalities during Ramadan fasting by patients with diabetes (78). That study found that deterioration in glycemic control during Ramadan was less prevalent among diet-controlled patients than those who used OHGAs (78).

The lack of proper education before Ramadan fasting may lead to adverse metabolic complications among fasting patients with diabetes. A qualitative study showed that some fasting patients experienced daily hypoglycemia in the afternoon because they had not adjusted their treatment schedule adequately [102]. That study also reported that other patients continued to inject short-acting insulin analogs at noon during fasting because they had not been told to stop their treatment or had not been made aware of the risk for hypoglycemia associated with not stopping. In addition, one patient erroneously doubled his SU dosage at noon assuming that would improve his glycemic control during night hours (102). Skipping the pre-dawn meal (*suhoor*) and taking SU medication before sunrise exposed fasting patients to a high risk for hypoglycemia. Furthermore, many patients insisted on not taking anything orally despite daytime hypoglycemia and did not seek any medical care if general malaise occurred as they did not wish to break their fast (102).

Body anthropometrics for fasting people with diabetes may affect the risk of developing HE during Ramadan. For example, Salti et al. (80) found that lower weight and smaller waist circumference were associated with increased risk for HE among fasting people during Ramadan. This could be explained by the fact that thin people have fewer glycogen stores than obese people, as obesity is associated with higher glycogen synthase enzyme expression and glycogen stores in adipose tissue (105).

### Sex Variations in HE

Sex was a variable that was expected to affect the occurrence of hypoglycemia and HE among fasting people with diabetes during Ramadan month. One study reported that females had a significantly higher frequency of severe HE and a lower mean number of fasting days than males during Ramadan month (61). This was supported by Masood et al. (97), who found that females with diabetes experienced hypoglycemia at a higher rate than males during their Ramadan fasting. This sex-based variation in hypoglycemia and HE could be attributable to differing habitual daily activities and routine work performed by males and females during Ramadan. For example, females (especially those taking care of their families) may spend more time and effort in housekeeping and working or child care, cooking, and worshipping, whereas males tend to spend more time in relaxation and sleeping during fasting days (106).

### Duration Since Diagnosis and HE

The duration since diabetes diagnosis is another factor that contributes to differences in hypoglycemia and subsequent HE among people with diabetes fasting during Ramadan. Early reports by Khalifa et al. (71) indicated that patients with a longer duration of diabetes had significantly higher frequencies of hypoglycemia during Ramadan (107). That study found that diabetes complications, including hypoglycemia, were significantly more represented among patients who had diabetes for more than 10 years compared with those that had diabetes for less than 10 years (32.2% vs. 12.1%) (107). This could be explained by reduced attention to the disease and its complications over time decreased effectiveness of medications used over a long time, or other social or socioeconomic factors affecting glycemic control among patients with diabetes.

### Seasonal Variations and HE

It is noteworthy that the majority of the identified studies were conducted during the summer season (studies published from 2010 to 2018). During summer, the duration of fasting is 12–21 h/day; these longer daytime hours mean that fasting during the summer season is expected to be associated with more HE than other seasons. Therefore, the season should be considered by patients when choosing to fast and by doctors in managing the patient’s blood sugar. Further, this seasonal variation in Ramadan fasting could be a subject for meta-regression and subgroup analysis. Such research would complement previous systematic reviews with meta- and subgroup-analyses that explored the impact of fasting duration as a significant moderator for the effect of RDIF on body weight and metabolic syndrome components in healthy people (108, 109).

### Effects of Medications

The included studies suggested that SU treatment was an inferior option in terms of the ability to maintain glycemic control and minimize the risk for hypoglycemia and subsequent HE during Ramadan fasting among patients with diabetes. SU agents have previously been associated with a higher risk for HE during Ramadan fasting than other OHGAs in patients with T2DM (32, 42, 50, 56, 65, 73, 83, 110). Further, SU use has been connected with patients breaking their fast because of intolerance to fasting, discomfort, and fear of hypoglycemia, which was not seen in patients using other OHGAs (e.g., vildagliptin) (77). However, one study (44) did not associate SU use (including gliclazide) with higher rates of HE compared with DPP-4 inhibitors as reported in some observational studies (64, 111). This could be attributed to the provision of individualized patient advice and education about dose adjustments used in these studies. Besides, another study supported the safety and effectiveness of SU treatment in lowering the risk for HE among adult males with T2DM. Zargar and colleagues (90) reported male patients under glycemic control with gliclazide MR 60 mg monotherapy could safely maintain the same degree of control by switching to an evening dose schedule during Ramadan, with a low rate of HE reported among these patients.

Conversely, other OHGAs showed superior effects, including MTF, SGLT2-I (that inhibit renal glucose reabsorption and increase renal glucose excretion), and repaglinide (short-acting insulin secretagogue that acts by binding to β-cells in the pancreas to stimulate insulin release). A recent expert panel statement on the use of SGL2-I highlighted the significance of the use of these hypoglycemic agents by patients with diabetes observing Ramadan. The expert panel discussed the efficacy and safety of SGLT2-I based on outcomes of recent clinical trials with SGLT2-I across the Middle East and Africa (MEA) region during Ramadan, with emphasis on hypoglycemia as a serious complication during Ramadan fasting. This statement aided physicians in the MEA region (which includes about one-fifth of the world’s Muslim population) regarding appropriate decision-making for patients during Ramadan (112). Repaglinide was found to effectively reduce the frequency of HE among patients during Ramadan fasting (81).

MTF is the preferred agent for managing patients with T2DM. MTF is associated with a 1%–2% reduction in HbA1c and carries a low risk for HE. Therefore, MTF is an attractive therapy for the majority of patients who decide to undergo fasting (113). If a patient is taking MTF three times daily, the midday dose may be omitted, and a larger dose may be taken in the evening (113–115). Those on a twice-daily regimen do not need to change administration times. However, patients who experience gastrointestinal side effects or HE symptoms should have their dose reduced. Overall, MTF has a low (but not negligible) risk for promoting HE (2).

### Combination of Medications

One study noted that the highest incidence of symptomatic HE during Ramadan was observed in T2DM patients treated with glipizide or glibenclamide, followed by those treated with glimepiride or gliclazide, with wide variation in the incidence of HE among those using different SU agents during Ramadan fasting (46). Further, the total number of breaking-the-fast events reported was highest among patients with T2DM treated with glibenclamide, with the highest incidence of severe HE (requiring medical assistance or not) occurring in this group (46). This finding contradicts the previous report by Belkhadir et al. (92), who found that glibenclamide was safe and effective in preventing hypoglycemia for patients with T2DM who fasted during Ramadan. Another study reported that the proportion of patients with at least one symptomatic HE was highest in the glimepiride group, followed by the glibenclamide group, and lastly, the gliclazide group treated with SU during Ramadan fasting (32). Similar findings were also reported for patients with T2DM fasting during Ramadan, where fewer patients on vildagliptin experienced one or more HE compared with commonly used individual SU agents (47). The highest proportion of HE was in the glibenclamide group, followed by gliclazide, glimepiride, and glipizide (47). Another study found that MTF with vildagliptin was efficient in lowering the incidence of hypoglycemia and severe HE among patients with diabetes treated with MTF and SU glinide (62) or MTF and SU gliclazide (59, 63).

The positive effect of vildagliptin in lowering HE is a known characteristic of this medication, with hypoglycemia rarely observed in clinical trials among patients with diabetes (116). The mechanism of sustained glucagon secretion during hypoglycemia by vildagliptin could be attributed to the ability of gastric inhibitory polypeptide triggered by vildagliptin that stimulates glucagon secretion during hypoglycemia (117). Dapagliflozin and liraglutide showed a superior effect in lowering HE, especially when combined with MTF compared with SU and MTF (57, 89). Further, the superiority of DPP-4 inhibitors (sitagliptin and vildagliptin) in lowering the HE was apparent among fasting patients with T2DM compared with those receiving SU treatment (7, 32, 47, 60, 73, 77). Similarly, liraglutide showed superiority in lowering HE among fasting patients with T2DM compared with SU treatment during Ramadan, suggesting liraglutide is a safe, effective, and well-tolerated choice when used as glucose-lowering therapy in patients with T2DM who choose to fast during Ramadan (50).

Vildagliptin is an attractive treatment option for patients with T2DM who are fasting during Ramadan (7). Vildagliptin can improve glycemic control by inducing glucose-dependent insulin secretion through inhibiting the DPP-4 enzyme, thereby enhancing the sensitivity and responsiveness of β- and α-cells to glucose (116, 118). The ability of vildagliptin to lower the risk for HE may be because suppression of both glucagon secretion and meal-dependent insulin secretion is glucose-dependent (119, 120). This favorable outcome observed during fasting is attributable to the suppression of inappropriate glucagon secretion during hyperglycemia and the enhancement of the glucose-dependent insulinotropic polypeptide (GIP)-mediated effect on glucagon, which results in a protection against hypoglycemia. The levels of both GLP-1 and GIP remain high during the inter-meal and overnight periods when hypoglycemia is more likely to occur (73). Sitagliptin, another DPP-4 inhibitor, also showed a superior effect in lowering symptomatic HE during Ramadan compared with SU agents in Muslim patients with T2DM (83).

The protective effect of canagliflozin (an SGL2-I) against HE among fasting patients with diabetes was further elaborated in the Canagliflozin in Ramadan Tolerance Observational Study. That study evaluated the tolerability of canagliflozin combined with MTF with or without DPP-4 inhibitors for the treatment of T2DM in patients fasting during Ramadan. The results showed that during Ramadan, patients on canagliflozin experienced fewer symptomatic HE compared with the SU group. Adjustment for medication was not needed for those on canagliflozin, whereas about 9% of those treated with SU adjusted their medication dose near the beginning of Ramadan (65).

Patients with T2DM treated with insulin showed variable hypoglycemia outcomes during their Ramadan fasting. While hypoglycemia may be predicted by insulin therapy (55), different reports indicated that combining basal insulin with incretin mimetics (which act like *GLP*-*1* receptor agonists) such as lixisenatide injection, showed better glycemic control and lower frequency and severity of hypoglycemia than combining insulin therapy with SU (79, 95). However, combining insulin glargine with the SU glimepiride was helpful in patients with T2DM who wished to fast during Ramadan, provided glimepiride was given at the time of breaking the fast and insulin glargine titrated to provide FBG >6.7 mmol/L (80). However, both rapid and long-acting insulin were significantly associated with hypoglycemic attacks (49), with HE highest in patients treated with insulin, followed by those treated with OHGAs with SU when compared with OHGAs without SU (51). A comparison between intensive insulin and basal insulin showed that the average number of HE was significantly higher in intensive insulin therapy than basal insulin therapy (54).

It is not recommended for patients with poorly controlled T2DM to fast because of the risk for dehydration associated with hyperglycemia. Clinical experience supports that patients with diabetes controlled by diet alone can safely fast. This is because fasting, especially the intermittent pattern followed during Ramadan, has many beneficial effects including metabolic and glucoregulatory mechanisms. Patients treated with OHGAs may fast if allowed by their physician; however, their regimen will need to be adjusted to reflect lifestyle changes. MTF and thiazolidinediones are the preferred hypoglycemic drugs because they are associated with a lower risk for HE compared with insulin secretagogues. Short-acting insulin secretagogues are preferable to SU. Insulin-treated patients may be treated similarly to patients with T1DM using a basal-bolus method or other regimens as recommended by their physician. Close follow-up and frequent monitoring are essential. Attention should be paid to the sunset fast-breaking meal, particularly in excessive intake of traditional sweets and sweetened drinks, as this can lead to uncontrolled postprandial hyperglycemia (28).

The variation in HE outcomes at the end of Ramadan could be attributed to various influencing factors that act simultaneously on shaping the effect of RDIF on HE and the related cardiometabolic risk factors. These factors could be classified into internal (patient-centered) factors and external (Ramadan month-related) factors. Among the internal factors, patients’ age, sex, time since diabetes diagnosis, pre-fasting control of blood sugar, commitment to the prescribed medications before and during Ramadan month, and adherence to prescribed diet and lifestyle modifications before Ramadan month. The external factors include fasting duration, climate conditions, working conditions, social activities, practicing religious rituals of Ramadan month, and the quantity and quality of foods consumed during the night hours (especially the pre-fasting meal or *suhoor*), with emphasis on the last time this meal was consumed before starting fasting. Other lifestyle changes accompanying Ramadan fasting month are thought to impact the extent to which RDIF affects cardiometabolic risk factors, including changes in sleep pattern/duration, diurnal exercise, changes in work schedule, nocturnal activities, and nocturnal light exposure (121). Further, the discrepancies in reported results concerning the effect of RDIF on HE could also be attributable to differing experimental and study designs. For example, RCTs allow control of confounding factors and minimize the risk of bias, therefore giving more robust and accurate measures for the targeted outcomes. However, observational designs, owing to their inherent limitations do not have such controls, which may result in less accurate outcome estimations (122).

### Insulin and Hypoglycemic Medications

During Ramadan, medications used by patients with diabetes may have to be adjusted. Patients who observe Ramadan fasting have to consider potential diabetic complications such as hyperglycemia, HE, dehydration, and ketoacidosis. Risk factors associated with fasting include the presence of co-morbidities and diabetic complications, advanced age, frailty, the risk for HE or a history of impaired HE awareness, living alone, T1DM, and pregnancy (2). Certain classes of glucose-lowering medications are associated with an increased risk for HE, such as SU agents and insulin. Besides, the largest dose of these medications should be taken in the evening, along with the large meal that is usually consumed at that time (*iftar*).

SU and insulin secretagogues are widely used during Ramadan. However, recent studies have highlighted an increased risk for HE during fasting among patients treated with insulin secretagogues (32, 42, 110). The risk for HE increases exponentially in older patients and patients with renal failure and medical conditions treated with SU (113). Long-acting agents such as glibenclamide (glyburide) should be avoided. The morning dose of shorter-acting preparations such as glipizide or gliclazide can be halved, and the evening dose kept the same. One study tried switching the timing of SU administration from morning to evening and found no effect on the rate of HE. A five-country observational study on SU treatment during Ramadan reported a 20% prevalence of HE. SU agents have a moderate to high risk for HE as they promote insulin secretion that is not glucose-dependent.

The variable effect of RDIF on the occurrence of HE among patients with diabetes could also be attributed to the different types of medications used in the management of diabetic patients fasting during Ramadan. Vildagliptin and gliclazide are among the commonly used medications that were effective in lowering the occurrence of HE among patients with T2DM fasting during Ramadan (64). Vildagliptin is an oral anti-hyperglycemic agent of the DPP-4 inhibitor class of drugs used for adults with T2DM. Vildagliptin enhances pancreatic islet cell responsiveness to glucose (123). Vildagliptin is a combination tablet containing MTF, whereas gliclazide is a SU insulin secretagogue that helps the pancreas make more insulin. The superiority of vildagliptin over other OHGAs was reviewed by Aziz (124), who found that in comparison with the OHGAs and SU agents (which carry a higher and significant risk for HE), DPP-4 inhibitors (e.g., vildagliptin) demonstrated a lower risk for HE during Ramadan fasting, with better patient compliance. This effect is supported by findings from many clinical observational and clinical studies in different locations around the world.

Decreased food intake is a well-known risk factor for HE among patients with diabetes (125). There are no reliable estimates concerning the contribution of HE to mortality in T2DM; however, it is thought that HE is an infrequent cause of death in this population (125). Rates of HE are lower in patients with T2DM when compared with T1DM (5), and are even lower in patients with T2DM treated with oral agents (5). Loke and colleagues investigated the effect of various risk factors on HE in patients with diabetes who fasted during Ramadan, and reported the rate of HE was 1.6 times higher during fasting compared with non-fasting periods (101). They observed that good metabolic control (<8%) and old age (>60 years) increased the risk ratio more than twice, and eating a meal before fasting reduced the risk ratio to less than half. However, the difference in that study was smaller than indicated in the EPIDAR study (5), which showed that fasting during Ramadan increased the risk for severe HE (defined as hospitalization due to HE) by 4.7-fold among patients with T1DM (from 3 to 14 events/100 people/month) and 7.5-fold in patients with T2DM (from 0.4 to 3 events/100 people/month). Severe HE was more frequent in patients in whom the OHGA or insulin dosage was changed and in those who reported significant lifestyle changes (5, 125).

About 1.5 billion Muslims globally are expected to observe Ramadan and refrain from food and drink from dawn to sunset. Even with the relatively large number of studies investigating RDIF and diabetes, the impact of RDIF on these patients is not fully understood. In particular, the effect of fasting during Ramadan on rates of HE in patients with diabetes is not known with certainty. There are a limited number of studies in this area, which warrants further longitudinal, controlled clinical studies. To our knowledge, this is the first systematic review exploring the impacts of RDIF on HE and related cardiometabolic risk factors in patients with diabetes. Although this systematic review could not reach definite conclusions because of conflicting findings and lack of control for confounding factors, we found no evidence suggesting a negative impact of observing RDIF on HE among patients with diabetes or other overt harms. Nonetheless, the authors emphasize the need to conduct further studies using larger datasets and controlled for more confounders, including meta-analyses of obtained data, and adjusted for different covariates using sub-group analyses.

The present review did not conduct a meta-analysis for the outcome of using different OHGA and insulin therapies in preventing HE during Ramadan. Nonetheless, the present findings regarding the positive effect of non-SU hypoglycemic medications during Ramadan (e.g., DPP-4 inhibitors) in lowering the incidence of HE among fasting people with diabetes mirrors the findings of a previous systematic review and network meta-analysis (126). That meta-analysis showed that DPP-4 inhibitors were associated with reducing the development of HE during Ramadan in both observational and experimental (RCT) studies. Further, the findings highlighted the importance of pre-Ramadan education as a beneficial tool in reducing HE during Ramadan.

### Limitations

The present systematic review entailed imitations related to the limitations of the sources studied. Differences in definitions and reporting of outcomes also made comparisons across studies difficult. Owing to the nature of Ramadan fasting, the studies were conducted in different geographical locations where daylight hours and climatic conditions differed, and fasting was observed under different conditions. The variable study designs and different demographic characteristics of study participants were further limitations that might have significantly impacted the accuracy of the conclusions from this systematic review. There were few studies with different patient subgroups and many studies used small sample sizes, which limited our ability to draw firm conclusions.

## Conclusions

Patients with T2DM may experience safer fasting and have a lower risk for acute complications if they comply with necessary adjustments in the timing and dosage of their medication. Better outcomes may be achieved if medication changes are accompanied by appropriate dietary and lifestyle changes. Hypoglycemia and HE are preventable for patients with diabetes observing Ramadan fasting. Mounting evidence suggests that combining MTF with OHGAs (e.g., a DPP-4 inhibitor and SGLT2-I with less reliance on SU) showed inferior effects in preventing HE among patients with diabetes that decided to fast during Ramadan. Ramadan fasting does not necessarily increase the burden of acute complications and cardiometabolic deteriorations unless the fasting patient does not comply with established medical and dietary guidelines for fasting during Ramadan. Patients with diabetes who decide to fast during Ramadan should be counseled regarding the importance of strict adherence to the adjusted dose and timing of their medication, adhere to healthy diet and lifestyle behaviors during Ramadan fasting, and be closely clinically monitored.

## Data Availability Statement

The original contributions presented in the study are included in the article/supplementary material. Further inquiries can be directed to the corresponding authors.

## Author Contributions

Conceptualization: MF and AB. Methodology: MF. Validation: MF and DA. Investigation: MF and DA. Resources: MF. Data curation: MF, DA, AY, and AS. Writing – original draft: MF, DA, and AB. Writing – review and editing: AB, AA, and MH. Visualization: MF, AB, AA, and MH. Supervision: MF. Project administration: MF and AB. All authors contributed to the article and approved the submitted version.

## Conflict of Interest

The authors declare that the research was conducted in the absence of any commercial or financial relationships that could be construed as a potential conflict of interest.

## Abbreviations

HE, hypoglycemic event; MTF, metformin; OHGAs, oral hypoglycemic agents; RDIF, Ramadan diurnal intermittent fasting; SGLT2-I, sodium-glucose co-transporter inhibitors; SU, sulfonylureas; T1DM, type 1 diabetes mellitus; T2DM, type 2 diabetes mellitus.
